# The Hedgehog-GLI pathway in embryonic development and cancer: implications for pulmonary oncology therapy

**DOI:** 10.18632/oncotarget.19527

**Published:** 2017-07-24

**Authors:** Leonel Armas-López, Joaquín Zúñiga, Oscar Arrieta, Federico Ávila-Moreno

**Affiliations:** ^1^ Universidad Nacional Autónoma de México (UNAM), Facultad de Estudios Superiores (FES) Iztacala, Biomedicine Research Unit (UBIMED), Cancer Epigenomics And Lung Diseases Laboratory (UNAM-INER), Mexico City, México; ^2^ Instituto Nacional de Enfermedades Respiratorias (INER), Ismael Cosío Villegas, Research Unit, Mexico City, México; ^3^ Instituto Nacional de Cancerología (INCAN), Thoracic Oncology Clinic, Mexico City, México

**Keywords:** lung cancer, epigenetics, lncRNAs, biomarkers, therapy

## Abstract

Transcriptional regulation and epigenetic mechanisms closely control gene expression through diverse physiological and pathophysiological processes. These include the development of germ layers and post-natal epithelial cell-tissue differentiation, as well as, involved with the induction, promotion and/or progression of human malignancies.

Diverse studies have shed light on the molecular similarities and differences involved in the stages of embryological epithelial development and dedifferentiation processes in malignant tumors of epithelial origin, of which many focus on lung carcinomas. In lung cancer, several transcriptional, epigenetic and genetic aberrations have been described to partly arise from environmental risk factors, but ethnic genetic predisposition factors may also play a role.

The classification of the molecular hallmarks of cancer has been essential to study and achieve a comprehensive view of the interaction networks between cell signaling pathways and functional roles of the transcriptional and epigenetic regulatory mechanisms. This has in turn increased understanding on how these molecular networks are involved in embryo-layers and malignant diseases development. Ultimately, a major biomedicine goal is to achieve a thorough understanding of their roles as diagnostic, prognostic and treatment response indicators in lung oncological patients.

Recently, several notable cell-signaling pathways have been studied based on their contribution to promoting and/or regulating the engagement of different cancer hallmarks, among them genome instability, exacerbated proliferative signaling, replicative immortality, tumor invasion-metastasis, inflammation, and immune-surveillance evasion mechanisms. Of these, the Hedgehog-GLI (Hh) cell-signaling pathway has been identified as a main molecular contribution into several of the abovementioned functional embryo-malignancy processes. Nonetheless, the systematic study of the regulatory epigenetic and transcriptional mechanisms has remained mostly unexplored, which could identify the interaction networks between specific biomarkers and/or new therapeutic targets in malignant tumor progression and resistance to lung oncologic therapy.

In the present work, we aimed to revise the most important up-to-date experimental and clinical findings in biology, embryology and cancer research regarding the Hh pathway. We explore the potential control of the transcriptional-epigenetic programming versus reprogramming mechanisms associated with its Hh-GLI cell signaling pathway members. Last, we present a summary of this information to systematically integrate the Hh signaling pathway to identify and propose novel compound strategies or better oncological therapeutic schemes for lung cancer patients.

## INTRODUCTION

Several findings have been integrated to define cancer as a group of malignant diseases of multifactorial and multigenic origin that are controlled by transcriptional control/modulation and epigenetic memory and/or reprogramming. Altogether, these mechanisms contribute to the induction, promotion, transformation and progression of malignant neoplasms, among which are tumors of epithelial origin, namely, carcinomas. Based on the “Hallmarks of Cancer” classification developed over a decade ago, we can now hierarchize the complex molecular events that determine the progression of the aforementioned processes, such as malignant neoplastic transformation and progression [1, 2]. During the last decade, extensive study of the epigenome has rendered a description of diverse epigenetic aberrations in different types of solid neoplasms, all of which have high incidences and mortality rates worldwide, including pulmonary, breast, colon and rectum, cervicouterine and ovarian carcinomas [3–6]. Nonetheless, the distinct mechanisms that are relevant in the transformation and progression of malignant neoplasms remain to be clarified.

It is therefore of particular importance to fully understand the early mechanisms of transcriptional and epigenetic regulation and modulation of gene expression in phenomena such as cell differentiation, cell-cycle control, apoptosis, autophagy, self-renovation, and the maintenance of stem-like cells of cancer, among other processes dictated in the hallmarks of cancer. Thus, the central role played by transcriptional-epigenetic control mechanisms in the development of complex and multifactorial diseases, such as malignancies, is evident [7]. One such example is the reversible DNA methylation process that is involved in embryologic as well as cancer progression mechanisms [8]. Similarly, reports from the last few decades have highlighted the biochemical process of post-translational modification of the histone code, which modulates, in a transitory and reversible manner, the expression level profiles of the cancer genome, whose molecular events affect malignant progression. It is interesting to note that some of these molecular events have a place in the physiological process of embryogenesis [9].

Based on this, transcriptional programming events and epigenetic reprogramming mechanisms actively intervene in erasing and writing DNA methylation patterns, as well as in the modification of histone codes through diverse embryo stages such as gastrulation and late embryonic development periods and postnatally; as such, they also participate during epithelial neoplastic transformation processes [10, 11]. This involvement results in the compromise of several intracellular signaling pathways, such as the Sonic Hedgehog (Hh) pathway, which is involved in many processes including the maintenance of physiological stem cells, embryogenesis and the transformation and progression of lung cancer, particularly non-small cell lung carcinomas [12].

As a result of the biological and physiological significance of the Hh pathway, the use of several inhibitors directed at members of the Hh pathway has been incorporated into the treatment of human carcinomas in the last decade; an inhibitor of particular importance is a monoclonal antibody directed at PTCH1 (anti-Patched1, m5E1 Developmental Studies Hybridoma Bank), which promotes inhibition at the membrane level. Meanwhile, small molecules such as GDC-0449 (Genentech), BMS-833923/XL139 (Exelixixs/Bristol-Myers Squibb), LDE-225 (Novartis), IPI-926 (Infinity Pharmaceuticals), and SANT (Sigma Aldrich), as well as AZD8542, a compound that acts as an SMO antagonist recently developed by AstraZeneca, are able to inhibit protein SMO activation at the cytoplasmic level. GANT58 and GANT61 compounds (Sigma Aldrich) are successful antagonists of transcription factors from the GLI family, which are the final effectors of the Hh pathway, at the nuclear level [13].

Nonetheless, the diverse set of mechanisms or regulation networks that genetically, epigenetically and transcriptionally regulate the Hh pathway remain to be elucidated, as does their probable impact on the control and/or promotion of multifactorial and/or complex diseases, such as carcinomas. The present review gives a recount of the principal experimental advances involved in the genetic-transcriptional and epigenetic control of the Hh signaling pathway involved in embryonic development processes and epithelial malignant transformation-progression. The main focus is put in Non-Small Cell Lung Cancer (NSCLC), which is a disease with high incidence and mortality rates even in patients with low, or even absent, exposure to environmental risk factors such as smoking, worldwide.

### The Hedgehog (Hh) signaling pathway in embryogenesis, stem cells and cancer

#### Hh signaling in embryogenesis

The first evidence relating the Sonic Hedgehog pathway to embryonic development was documented in the 1980s with a study by Nusslein-Volhard and Wieschaus who identified lethal genes during the embryonic development of *Drosophila melanogaster* by directed mutagenesis assays. They attained evidence of a loss of the ventral and bilateral pattern during development. It is worth mentioning that the name of the pathway, Hedgehog, originated from the short, pointed phenotype of the cuticle raised by the mutated Drosophila larvae, which has been observed in different versions of the Hh-GLI pathway, such as Sonic Hedgehog (SHH), Indian Hedgehog (IHH), and Desert Hedgehog (DHH), all of which develop a phenotype similar to the spikes of a hedgehog [14]. Additionally, it is important to recognize how some of the Hh-GLI1 pathway members have been implicated as key mediators in fundamental cellular processes for embryonic development in vertebrates, acting as morphogenic factors promoting dose-dependent induction differentiation and/or cell fate. They also have an effect as mitogens, controlling cell proliferation, survival, and organogenesis in different anatomical regions of vertebrates and, as an induction signal of the ventral neural tube, development of the anterior-posterior axis of extremities and somatic ventral structures [15, 16].

It has been recognized that the secretion and signaling function of the Hh pathway proteins are evolutionarily conserved in *Drosophila melanogaster* and superior vertebrates. Nonetheless, in mammals, three different genes have been described: SHH, IHH and DHH, each with a different spatial and temporal distribution [17].

Although the Hh signaling pathways are involved in diverse development stages, one of them is fundamental in craniofacial morphogenesis. This is an intricate process that begins with the development of the head primordials, which are involved in different organization centers located in the neural ectoderm, the cranial neural crest, and the axial mesoderm. The differentiation and spatial pattern in this bone region must occur prior to tissue fusion-integration [18].

In this sense, the active molecule SHH has been designated the involved morphogen in the signals for axial and dorso-ventral definition of craniofacial development and of lateral member development [19–21]. Deregulation events of the Hh pathway have been linked to a broad set of pathological craniofacial manifestations including Cyclops, hypertelorism, and holoprosencephaly (HPE), among others [22, 23]. It is important to highlight that the most studied morphogen factors, Indian and Sonic Hedgehog, have been fully linked to the formation of cartilaginous tissue, the axial, appendicular and facial axis bone pattern of all the human skeletal system [24, 25]. The roles of the Hh signaling pathway in embryogenesis, craniofacial ossification, and limb development have been broadly documented throughout the XXI century. As such, advances in experimental research of final effector genes involved in this pathway stand out for their localization at the cell nuclei level, their transcriptional level function has been found to be based on transcriptional factors belonging to the GLI family. Such is the case of GLI-1, which is key for understanding the transcriptional and genetic expression modulation mechanisms involved in the physiological maintenance and development of superior organisms as well as in pathophysiology.

**Figure 1:**
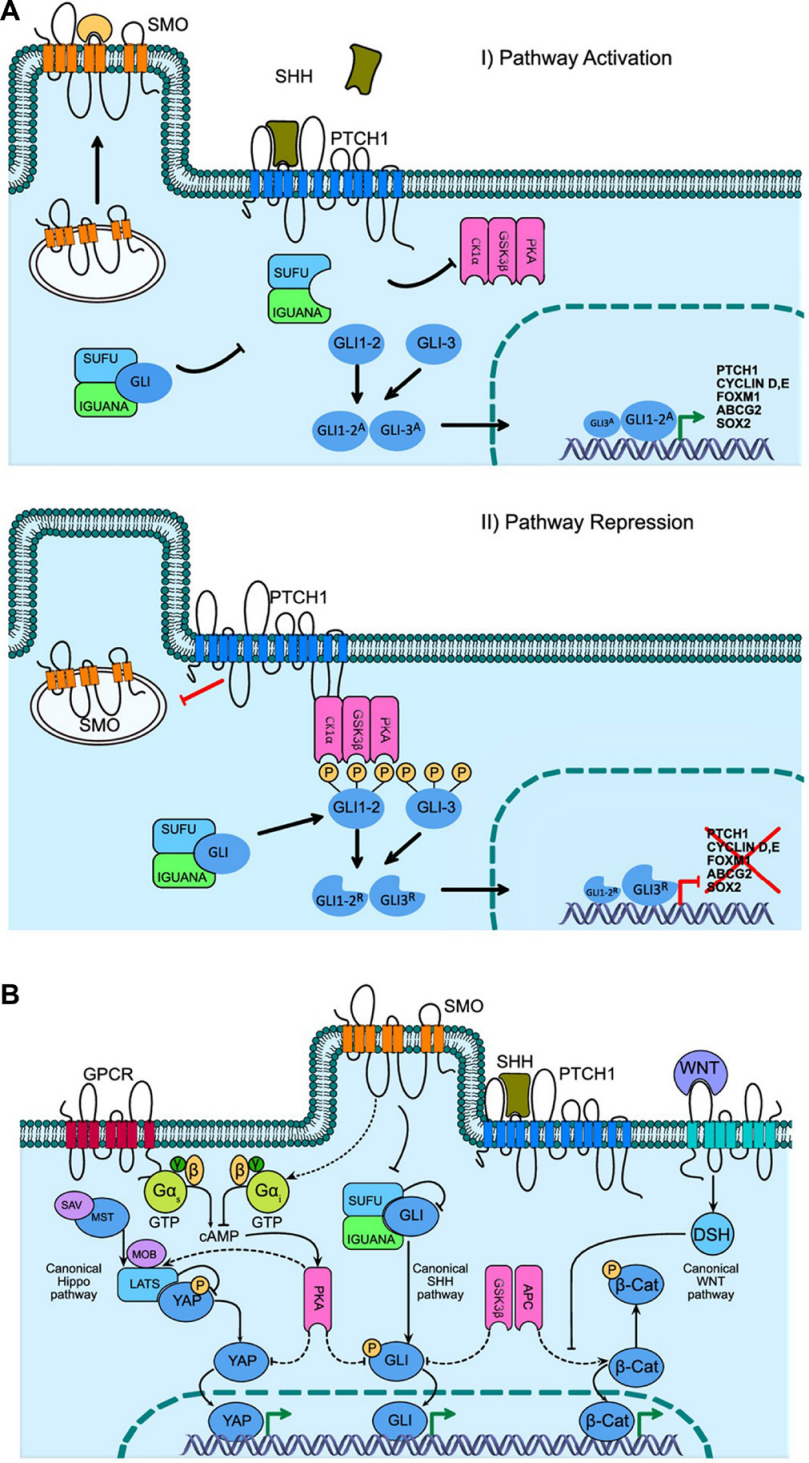
Hedgehog signaling pathway Gene activation, repression and cross-talk with other key self-renewal pathways (**A**) Activation of the Hh pathway begins with the binding of the SHH ligand to the membrane receptor PTCH1, removing the catalytic inhibition on SMO and promoting its displacement to the cell membrane for a posterior interaction with an endogenous ligand (a GTPase type protein has been proposed), promoting the nuclear translocation and transcriptional activity of GLI1^A^. The absence of SHH maintains the interaction of PTCH1 on SMO, contributing to the inhibition by blocking its displacement to the cell membrane, and promoting GLI proteolysis, which translocates as GLIR, this in turn modulates the transcriptional repression of, among others, PTCH1, Cyclin D and E, FOXM1, ABCG2, SOX2. (**B**) The cross-talk between renewal and cancer pathways is mediated by the PKA and GS3Kβ kinases, with the canonical Hippo PKA being able to phosphorylate the LATS and MOB proteins by carrying a cascade of phosphorylation to YAP, inactivating its nuclei translocation capacity. While the WNT pathway, through GS3Kβ in conjunction with APC phosphorylates β-Catenin, promoting proteasome degradation and inhibition of translocation to nucleus, while equally PKA and GS3Kβ phosphorylate members of the GLI family, preventing its translocation to nucleus and altering its functional cell signaling capacity.

Under normal, physiological conditions such as embryogenesis, the Hh proteins are synthesized as precursor molecules with C-terminal and N-terminal domains, the latter of which is rescinded and recognized as the signaling HhN (N-terminal Hh). Meanwhile, the C-terminal domain of the Hh polypeptide catalyzes the transfer of a cholesterol molecule to the signaling N-terminal domain. As a result, the cholesterol establishes an association with the lepidic structure of the cell membrane, facilitating the final processing stage, the addition of a palmitate molecule to the signaling HhNP domain, completing the biochemical process necessary for activation of the ligand-dependent pathway [26, 27]. During embryonic development, several autocrine and paracrine processes have been detected based on the cell secretory activity of the Hh pathway. Figure 1A shows the activation *vs* repression *momentums* of the Hh signaling pathway, the processes of which are initiated through the union of the Hh ligand and the receptor protein Patched1 (PTCH1), causing activation. This protein consists of 12 trans-membrane domains and can exert a catalytic inhibition of SMO, a trans-membrane protein of 7 domains, achieving a delocalization on the cell surface in response to an inhibition-activation state, scrolling from endosome structures in the cytoplasm to ciliary structures at the cell membrane level. Additionally, the role of another, yet unknown, intermediary molecule has been suggested, which probably acts as an SMO agonist aiding its transport towards PTCH1 in the membrane [28, 29].

The final effector route of the signal transduction of the Hh pathway is mediated by the molecular balance between the activating form (GLI^A^) and the repressor form (GLI^R^) of the coding gene Glioma-Associated Oncogene Homolog 1 (GLI-1), a member of the zinc-finger transcription factor family, which interact with promoter and regulatory sequences of DNA. In *Drosophila*, it has been previously described that the activation of GLI transcription factors occurs through the Hh signal and the protein complex Costal2 (cos2), Fused (fu) and Fused-suppressor (SUFU), up to the activation of transcription factor Ci (*Drosophila*), a GLI-homolog (Figure 1A). It is important to note that although in superior organisms the cos2 and fu factors are not conserved, SUFU has been well conserved and plays a fundamental role in cell signaling transduction pathways in mammals. It is also important to mention that while the Hh signaling process is generated from cell compartments localized in portions of the membrane temporarily found in cilia structures, the process has been described in non-ciliated cells where SMO and other coupling proteins are requisite for the activation of transcription factors of the GLI family (Figure 1A) [30, 31].

In this regard, 3 members of the GLI transcription factors family have been described, and all have a functional zinc-finger domain. Of these, GLI-1 (Chr:12q13.3) and GLI-2 (Chr:2q14.2) mainly have a transcriptional-activator function, while GLI-3 (Chr:7p14.1) acts as a transcriptional repressor factor [32]. A balance between these three factors has been proposed as a molecular code that permits the regulation of cell differentiation fate and compromise and participates in the maintenance of stem cells, which could have implications for cancer development. For example, stem cell niches have a higher expression of GLI-1, while differentiated cell populations highly express GLI-3. In the same manner, the histopathological progression of cancer has a markedly higher expression of GLI-1, unlike early disease stages, which show a predominance of GLI-3 expression over GLI-1 [33]. It is important to comment here that GLI-1 expression is dependent on activation of the Hh pathway, as, in absence of the SHH ligand, the PTCH1 protein exerts its inhibitory function on SMO, and GLI-1 is proteolytically processed until the repressor form, GLI^R^, is formed. This result yields the protein GLI-3, which contributes to the transcriptional repression of target genes of the Hh pathway. The union of the SHH ligand to PTCH1 removes its inhibition on SMO, causing the generation of the active form GLI^A^ and promoting, in an important manner, the presence of GLI-2, thus transcriptionally activating the expression of target genes of the Hh pathway [34]. It is also known that the GLI activation process is positively and negatively regulated through phosphorylation, which occurs through the intermediary molecules SUFU, IGUANA, PKA [35], GSK3B (Glycogen synthase kinase 3 beta), and CK1a (Casein Kinase 1 Alpha), among others (Figure 1A) [36, 37].

This information makes clear that the Hh signaling pathway is crucial not only during embryonic and postnatal development but also in the biology of malignant transformation. In regard to the first, an imbalance in the activation of the pathway can lead to structural and phenotypic malformations in higher organisms, such as facial complications, holoprosencephaly, microcephaly, cyclops and cleft palate. [23, 38]. Meanwhile, in the adult stage, constitutive activation or modulation of the Hh pathway is involved in tissue homeostasis and contributes to the renovation and repair process of tissues as well as the maintenance of physiological stem cells [39, 40].

### Hh in stem cells

Signal transduction through the Hh pathway regulates diverse precursor proteins in a tissue-specific manner, including the cerebellum, brain cortex and central nervous system. It also regulates the function of neural stem cells in the neurogenic niche of the hippocampus and the subventricular zone of the anterior brain [41, 42]. Additionally, the activation of this pathway promotes the regeneration and expansion of hematopoietic stem cells in bone marrow [43]. It has also been seen that epithelial stem cells remain in a quiescent state partly due to their limited GLI-1 expression in epithelium; in this regard, the limited self-renovation and generation of epithelial stem cells is partly explained by the dependence of these processes on the Hh pathway. As previously described in the adult brain, cells with a neural stem phenotype can increase the number and size of structures named neurospheres as a consequence of the Hh cell signaling pathway activation. This occurrence raises the possibility of manipulating GLI-1 expression, and therefore the Hh cell signaling pathway activity, in order to promote the genesis of stem cells in the treatment of neurological degenerative diseases [44].

It has also been proven that sustained activation of the Hh pathway in adult tissues, as well as the increased expression of GLI transcriptional factors in -epithelial tumors- carcinomas, maintains a close resemblance and molecular identity to the cancer stem cell phenotype. This phenomenon also increases the functional capacity of tumor self-renovation, invasion, metastasis and malignancy, which has been seen in animal *in vivo* models, as well as in solid tumors derived from patients with diverse epithelial malignancies, including breast, pancreatic, skin and brain cancer [45–48].

SH-dependent stem-capable cells could also be responsible, as previously proposed, for the treatment failure and relapse seen in different oncologic therapeutic schemes through cellular mechanisms of immune evasion, tumor metastasis, and resistance to either pharmacological or targeted therapy [49]. This novel area of research is therefore expected to produce knowledge to better understand the induction-promotion-progression processes involved in cancer in the context of the current therapeutic, diagnostic and prognostic tools for cancer patients [50].

The role of cross-talk between cell signaling pathways that promote mechanisms of renewal and cancer has also been previously described, where the functional participation of the enzymes kinases PKA and GS3Kβ has been involved.

One such case is the crosstalk of the Hippo canonical pathway, where PKA is able to phosphorylate the LATS and MOB proteins by promoting the cascade of phosphorylation through the YAP protein, where phosphorylated YAP is inactive and unable to be translocated to the nucleus, which promotes the expression of genes involved in the regeneration and repair process of stem cells in cardiac muscle and epithelial dermis tissues [51, 52].

Meanwhile in the WNT pathway the enzyme GS3Kβ together with APC phosphorylate β-Catenin, promoting its inactivation and degradation by a proteasome pathway, widely reported in different malignant tumors among others, colon cancer and gastrointestinal cancer [53, 54].

In the same way that the enzymes PKA and GS3Kβ are responsible for phosphorylating the proteins GLI, YAP and β-Catenin, preventing their process of nuclear translocation, being a consequence of the cross-talk between the self-renewal and cancer cell signaling pathways [53, 55] (Figure 1B).

### Hh in cancer progression

**Figure 2:**
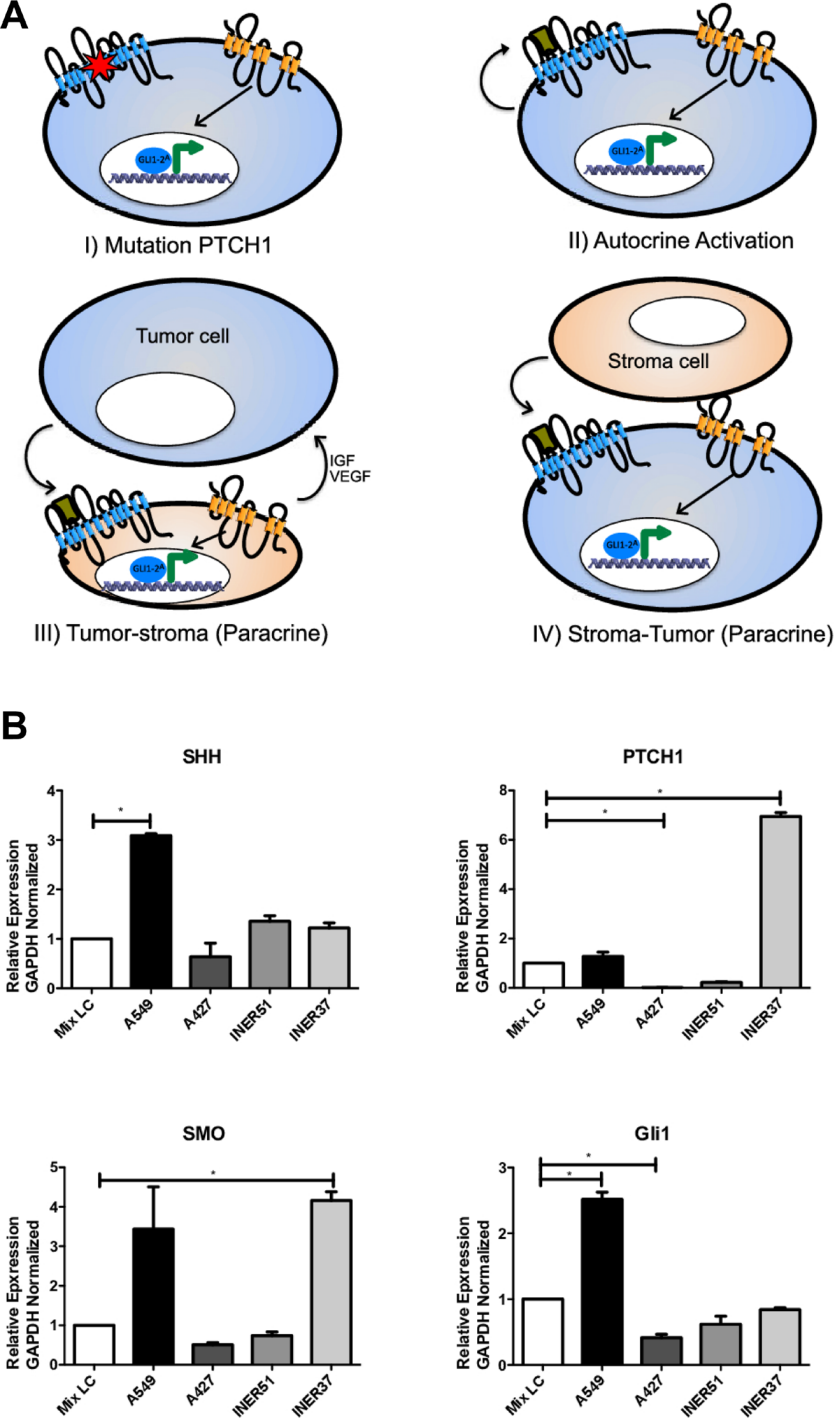
Over-activation mechanisms of the Hh pathway in cancer, and gene expression profile in lung cancer cell lines (**A**) Schematic representation of autocrine and paracrine activation of the Hh pathway: i) Ligand-independent: Constitutive activation of the pathway by mutation of the PTCH1 gene, promoting the maintenance of neoplastic cells; ii) Ligand-dependent: Autocrine activation occurs when neoplastic cells secrete their own ligand and achieve self-activation; iii) Paracrine activation: Neoplastic cells produce a ligand capable of activating stromal cells, which in turn secrete growth factors which maintain neoplastic cells; iv) Stromal cells secrete Hh ligand, promoting the activation of the pathway in neoplastic cells. (**B**) mRNA expression patterns for genes which code for Hh members, gene expression analysis in adenocarcinoma-type lung cancer cells A549, A427, INER51 and INER37(123-125). A549 cells possess higher level of expression of SHH, PTCH1, SMO and GLI-1, confirming previous reports where A549 remains the most adequate cellular model for the study of the SHH pathway in pulmonary, epithelial neoplasms (126). Expression analysis obtained using real-time PCR platform LightCycler 480 (Roche, Mannheim, Germany), with UPL-type specific hydrolysis probes (Roche, Germany) and normalizing expression levels through detection of endogenous gene GAPDH, taking as basal expression level the pooled data of mRNA of the 4 lung cancer cell lines analyzed.

The first description of the role of the Hh pathway in neoplastic transformation arose from observations in central nervous system tumors, glioblastomas, and melanomas associated with a genetic amplification of GLI-1 [56]. Later, PTCH1 mutations were associated with over-activation of the Hh pathway in basal cell carcinoma and medulloblastoma [57, 58]. In this regard, there are 4 possible models that attempt to explain the activation in physiological, normal conditions versus the over-activation of the Hh pathway in cancer (Figure 2A).

### Ligand-independent activation (I: Dependent on mutation)

Mutations in the gene that codes for the protein or the trans-membrane receptor PTCH1 have been previously described in patients with Gorlin syndrome, which is associated with a higher risk for harboring Basal Cell Carcinomas (BCC) [59]. This observation suggests that BCC tumors, melanomas, and medulloblastomas, among other solid neoplasms derived from the ectoderm, can favor over-activation of the Hh signaling pathway when in the presence of PTCH mutations or low-level expression of PTCH1. In this scenario, the pathway intermediates SMO and GLI-1 would contribute to the autonomous maintenance of epithelial malignant neoplasms [56, 57].

### Ligand-dependent activation (II: Autocrine)

High-level production of the SHH ligand has been described as a tumor mechanism of autocrine over-activation of the ligand-dependent Hh signaling pathway [60]. This mechanism has been described in several tumors of epithelial origin, such as small cell lung cancer (SCLC), pancreatic, colon, and prostate cancer, and glioblastomas and medulloblastomas, all of which originate from specialized epithelial tissues [61–65].

### Ligand-dependent activation (III, IV: Paracrine)

During embryogenesis, stromal cells intervene and secrete SHH ligands on neighboring epitheliums, acting as receptor cells [66]. Recent studies demonstrate that the tumor microenvironment promotes the paracrine activity of the Hh pathway, acting in a reciprocal manner between tumor and stromal cells [67–69]. Additionally, it has been shown that in neoplastic events such as B cell lymphoma and multiple myeloma, production of the SHH ligand originates mainly from stromal cells derived from bone marrow, the spleen and lymph nodes, causing an activating paracrine effect on neoplastic cells [67].

The paracrine capacity of solid tumors has been proven in pancreatic and colon cancer, along with xenotransplanted cell lines, on the expression profile of target genes of the Hh pathway in stromal cells [68–70], as well as the conserved genetic expression of the Hh pathway members in lung cancer cells of epithelial origin (Figure 2B). It is therefore evident that paracrine and/or autocrine activation have a determinant role in the progression of malignancies of epithelial origin, which has in turn consolidated the therapeutic pertinence of inhibiting the Hh pathway in patients with different types of malignancies derived from epithelial tissue.

### Hh pathway inhibition in lung oncology therapeutics: Cancer compounds and drugs

**Figure 3:**
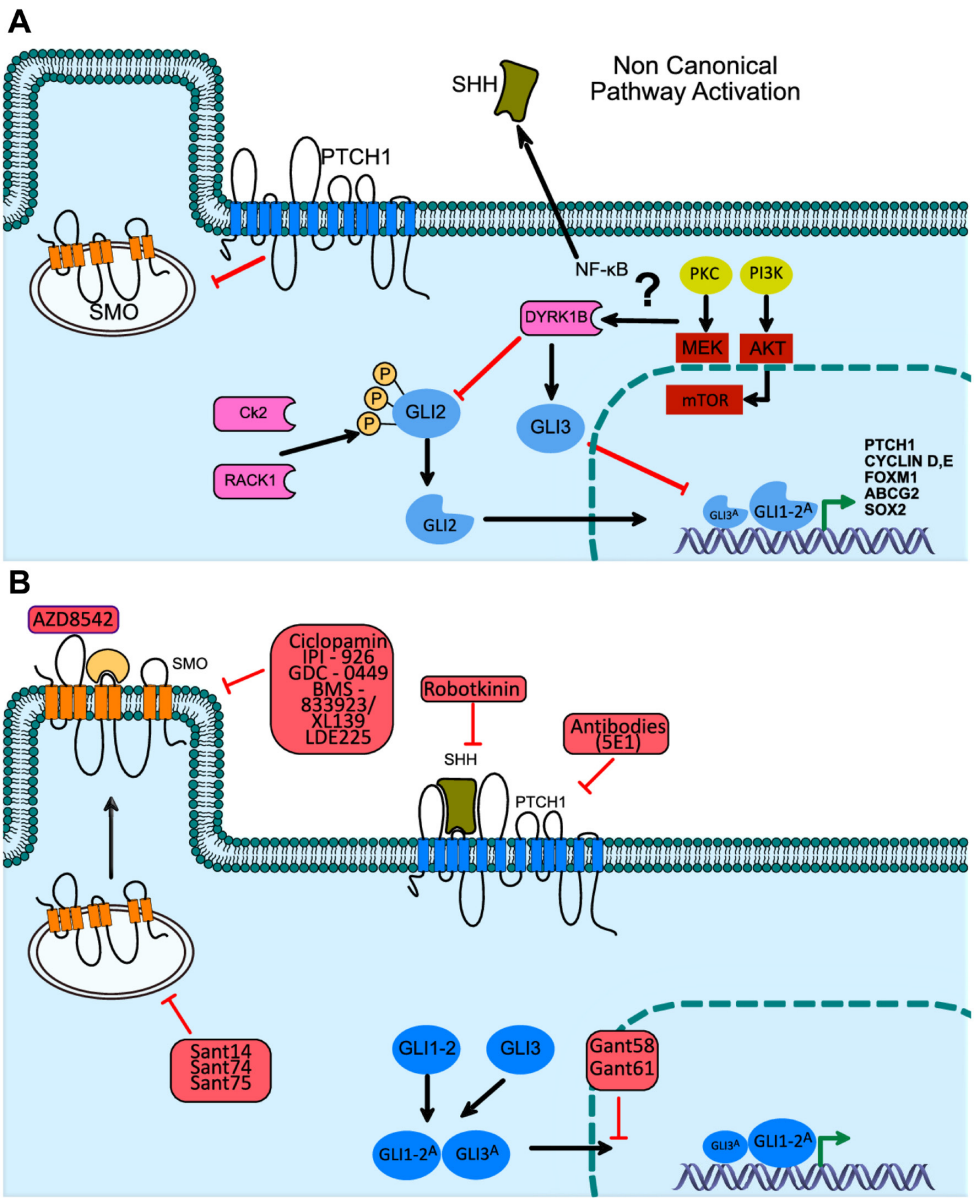
Non-canonical activation of the Hh pathway, through DYRK1B kinase, Hh signaling pathway inhibitors in medical oncology (**A**) The non-canonical activation of GLI-1/2 through Rack1 kinase has recently been described, bypassing pathway activation deficiencies through the binding of the SHH ligand to the PTCH receptor. (**B**) Through the canonic activation mechanism of the Hh pathway, diverse strategies have been designed to achieve the selective inhibition of the SMO protein. The patented molecules have been employed to generate a downstream inhibition of the Hh pathway, through the use of these compounds new pre-clinical and clinical trials have been developed, contributing to the survival increase of patients diagnosed with diverse epithelial type neoplasms.

Different transcriptionally regulated cell signaling pathways are in convergence or cross-talk in different cellular biological processes, among which RAS and Hh signaling pathways have been highlighted to promote tumorigenic processes. Of these, 5 members of the DYRK (Dual-specificity and Tyrosine (Y) -regulated kinase family of kinases) family of proteins have been highlighted for their capacity as regulatory enzymes [71]. DYRK1B, for example, is involved in the transcriptional regulation of the Hh signaling pathway, preventing its autocrine (canonical) activation, however this is likely to occur in the non-canonical pathway by inhibiting GLI2^A^ activity, increasing GLI3^R^. However, during this process an increase of the HH ligand (paracrine activation) has been described [72], whose effect is mediated by the previously unknown capacity of DYRK1B to activate the PI3K / mTOR / AKT pathway. This pathway is known to stabilize to the GLI protein family members [73], probably representing a novel target gene in the oncological therapy and Hh-GLI signaling pathway (Figure 3A).

The over-activating mechanisms that work through a non-canonical HH-GLI1 pathway (Figure 3A) have been used as targets for therapeutic strategies in epithelial BCC or medulloblastoma, both of which depend on genetic mutations of PTCH1 for activation, and as such may encounter successful inhibition with specific compounds (Figure 3B). Even so, the combination of specific inhibitors of the Hh pathway can be more efficaciously used in tumors with an autocrine or paracrine ligand-dependent activation. In this regard, Hh pathway antagonists include the SMO and GLI-1 proteins as the main targets for therapeutic schemes against cancers of epithelial origin. Nonetheless, most efforts have been focused on the pharmacological inhibition of the SMO protein, while the development of GLI inhibitors has additional and relevant merit, given that these proteins can be activated by mechanisms that may be dependent or independent of the SHH ligand through a non-canonical pathway of GLI-1 activation (Figure 3A, 3B). To this day, two SMO protein inhibitors have received approval from the Food and Drug Administration (FDA) for BCC treatment, namely, LDE225/Sonidegib and GDC-0449/Vismodegib, while at the same time, several clinical trials have evaluated their efficacy as directed therapy for neoplasms of the prostate (phase II), pancreas (phase II), breast (phase I), and lung (phase I/II) [13, 74].

We now describe the role of some of the molecules employed as Hh pathway inhibitors. During the last decades several drugs and compounds considered as teratogens, some of them of vegetal origin, have been used against cancer cells. Rationale relies on the fact that they can block vital cellular functions, thus causing defects at birth in animals that consume them as they can target embryonic cell signaling pathways. However, such teratogens also succeed in inhibiting the growth of neoplastic cells due to their ability to inhibit active embryonic cell signaling pathways. Currently some teratogenic compounds have been chemically modified reducing their adverse effects on patients, promoting greater bioavailability and increasing their therapeutic efficacy in combination with oncological drugs for cancer patients [75]. One such example is cyclopamine, an alkaloid compound isolated from *V. californicum*, which was the first identified inhibitor of the Hh pathway. Cyclopamine is able to successfully bind to the transmembrane domains of the SMO protein, impeding its activation and thus inhibiting the signaling cascade [76–78]. Even though it has low affinity and poor pharmacokinetic bioavailability, cyclopamine is able to reduce tumor size *in vivo*, and thus it has been used therapeutically despite the side effects seen in animal models [79]. Due to the previously mentioned limitations, new compounds have been synthesized that are derived from cyclopamine but have higher affinities and stabilities with reduced side effects [80].

In SCLC tumors, the canonical cell signaling of Hh has been therapeutically approached in the same manner, based on the inhibition of SMO activation, by reducing the stabilization and activation of the GLI1 transcription factors family. Although there are currently no reports describing additional mechanisms on the inhibition of SMO-GLI in SCLC tumors, SMO antagonists, such as erismodegib and vismodegib, continue to be employed as they block cell proliferation and increase apoptosis. Even though, the exact mechanisms involved in the antitumor effects seen through SMO inhibition, have not been fully elucidated [81, 82]. The highlights of some of the main SMO inhibitors used in current oncology therapy are shown in Figure 3B.

### GDC-0449 inhibitor

This compound was the first SMO inhibitor, developed by pharmaceutical Curis and Genetech, and it has been successfully employed in clinical oncology for treating medulloblastoma and BCC by inhibiting activation of the Hh pathway [83, 84]. Phase I trials provided evidence as to the efficacy of this compound in a 68-patient cohort study that included subjects who had a BCC diagnosis with over-activation of the Hh pathway. Thirty-three patients with advanced metastatic BCC who received GDC-0449 had a partial response to treatment, while complete tumor remission was seen in 2 patients [85]. However, toxicity and adverse events were reported, most frequently impaired or loss of taste, along with hair and weight loss. Skin biopsy analysis showed a decrease in the expression of mRNA for GLI-1 posterior to treatment with GDC-0449. In addition, tumor regression in a case of metastatic medulloblastoma with no response to multiple treatment schemes based on carboplatin, etoposide, cyclophosphamide, and vincristine was seen after treatment with GDC-0449. The male 26-year-old patient had rapid tumor regression, with a tumor molecular analysis which showed a successful inhibition of the Hh pathway [86]. In this sense, it is worth mentioning that GDC-0449, which has been FDA approved since January 2012, constitutes the first pharmacological inhibitor of the Hh pathway indicated for any type of neoplasm of epithelial origin. It has been commercialized under the name of Vismodegib as a second-generation compound derived from cyclopamine. It has since been used to treat patients with BCC tumors who are not candidates for radiotherapy or curative surgery [87].

### IPI-926 inhibitor

This inhibitor was created by Infinity Pharmaceuticals and is currently commercialized under the name Seridegib. It is a derivate of cyclopamine and potently inhibits SMO, thus efficiently reducing Hh pathway activation and in turn decreasing tumor growth in *in vivo* models. When administered in doses of 40 mg/kg it achieves recurrence-free tumor regression at 30 days [88]. IPI-926 has additionally been demonstrated to reduce neoplastic growth in xeno-transplanted primary tumors derived from chondrosarcoma patients who underwent curative surgery. A reduction in the tumor mass is partly explained by the decrease in the expression of GLI-1 and PTCH1 when compared to control groups. Meanwhile, in animal models of pancreatic cancer, the administration of IPI-926 induces a greater anti-tumor effect when administered along with gemcitabine, the cytotoxic activity of which relies on the inhibition of DNA synthesis, promoting cell death by apoptosis [89].

### BMS-833923/XL139 inhibitor

Commercialized by Exelixis/Bristol-Myers Squibb, this SMO inhibitor has a higher oral bioavailability in pharmacodynamic models [90] and has been proven to decrease the expression of GLI-1 and PTCH1 in wild-type cells as well as in SMO mutated cells. This decrease in turn inhibits the Hh pathway in a dose-dependent manner, avoiding the clonal expansion of multiple myeloma (MM) precursor cells. Similarly, the efficacy of the compound has been proven in esophageal adenocarcinoma cell lines, decreasing cell proliferation and increasing apoptosis [91].

### LDE225 inhibitor

This agent is known as Erismodegib/Sonidegib/Odomzo, and it is an SMO inhibitor with high oral bioavailability. It is commercialized by Novartis pharmaceutical and has been shown to increase apoptosis and to modulate cell cycle control in medulloblastoma [92]. LDE225 represents the second Hh pathway inhibitor approved by the FDA since July 2015 for treating BCC tumors in adult patients who are not candidates for radiotherapy or curative surgery. Additionally, it has been shown that LDE225 inhibits the epithelial-mesenchymal transition (EMT) phenomenon, as well as invasion processes, in prostate cancer and glioblastomas [93, 94].

### AZD8542 compound

An SMO antagonist recently developed by AstraZeneca, AZD8542 has proven an efficacious compound that blocks the paracrine signaling mechanisms dependent on stromal cells, obtaining an efficient treatment for pancreatic cancer stem cells with high expression levels of the SMO receptor and low levels of HH ligand. Treatment with AZD8542 has been able to decrease the expression of SMO and inhibit tumor growth in human pancreatic stellate cells (HPSCs) [95].

Small molecules that have the ability to antagonize SMO and GLI-1 in humans have been synthesized with the intention to experimentally and clinically evaluate the efficacy of the participation of diverse members and mechanisms of the Hh pathway. In this regard, the SMO antagonist molecules, SANT, possess the ability to modulate the Hh pathway in BCC tumors, affecting the expression of its genetic targets such as PTCH1 or its final effectors such as GLI-1 [96]. Other small molecules that antagonize GLI-1 (GANT58 and GANT61) have higher specificities and efficacies by decreasing the binding capacities of transcriptional activating factors GLI-1 and GLI-2 to promoter sequences of their target genes. This in turn promotes the inhibition of cell proliferation and increases cell death, reducing the expression of PTCH1 mRNA in pancreatic cancer cells [97, 98].

Other molecules able to inhibit the Hh pathway have been reported. These compounds block the Hh pathway through the union of the ligand Robotnikinin, a compound able to bind to the SHH ligand and block its union to PTCH1 in medulloblastoma, BCC, pancreatic and prostate cancer [99]. Meanwhile, blockage of the Hh pathway can also be achieved through antibodies that impede the interaction of the SHH ligand with PTCH1. Such is the case of anti-Patched1 (5E1 Developmental Studies Hybridoma Bank), which is commercially available for application and preclinical validation studies [100, 101]. A comprehensive summary of the abovementioned information may be consulted in Table 1.

**Table 1:** Inhibitor compounds

Inhibitor Name	Commercial name(s)	Company	FDA	IC50	Clinical Trial	Cancer Type	Reference
GDC-0449	Vismodegib/ Erivedge	Roche/Genentech/Curis	approved	0.5 uM DAOY cells	Phase 0, I, II, IV	BCC Medulloblastoma Prostate Esophageal Gastric Myeloid Leukemia Pancreatic Lung	72–76
IPI-926	Saridegib/Sadegib	Infinity Pharmaceuticals/Novartis	unnaproved	9 nM in C3H10T1/2 cells	Phase I, II	Mielofibrosis Chondrosarcoma Esophageal	77–78
BMS-833923/XL139	-	Exelixis/Bristol-Myers Squibb	unnaproved	10 um OE19 and OE33 cell lines	Phase I, II	Gastrointestinal Multiple myeloma Lung Gastrointestinal Multiple myeloma Solid tumours Myeloid leukaemia	79–80
LDE225	Erismodegib/Sonidegib/Odomzo	Novartis	approved	12 um A2780ip2 cell line	Phase I, II, III	Esophageal Ovarian Hepatocelular Prostate	81–83
AZD8542	-	AstraZeneca	approved	2.9 nM C3H10T1/2 cell line	Phase II	Pancreatic	84
SANT	-	SIGMA	unnaproved	20 nM in NIH 3T3 cell line	-	Basal cell Pancreatic Prostate Lung	85
GANT58	-	SIGMA	unnaproved	5 uM in NIH 3T3 cell line	-	Prostate Pancreatic Lung Glioma	86
GANT61	-	SIGMA	unnaproved	5uM in NIH 3T3 cell line	-	Prostate Pancreatic Lung Glioma	87

It is therefore evident that the study of the many mechanisms that accompany the expression and functional participation of the Hh pathway is crucial in order to contextualize the presence and application of its biological and physiological impacts as well as its clinical potential in patients. The study of the transcriptional regulation and epigenetic mechanisms of the Hh pathway members has thus become of utmost importance due to their molecular and clinical relevance.

### Epigenetic regulation of the Hh pathway: implications for pulmonary oncology therapeutics

#### Epigenetic profile of the Hh pathway

**Figure 4:**
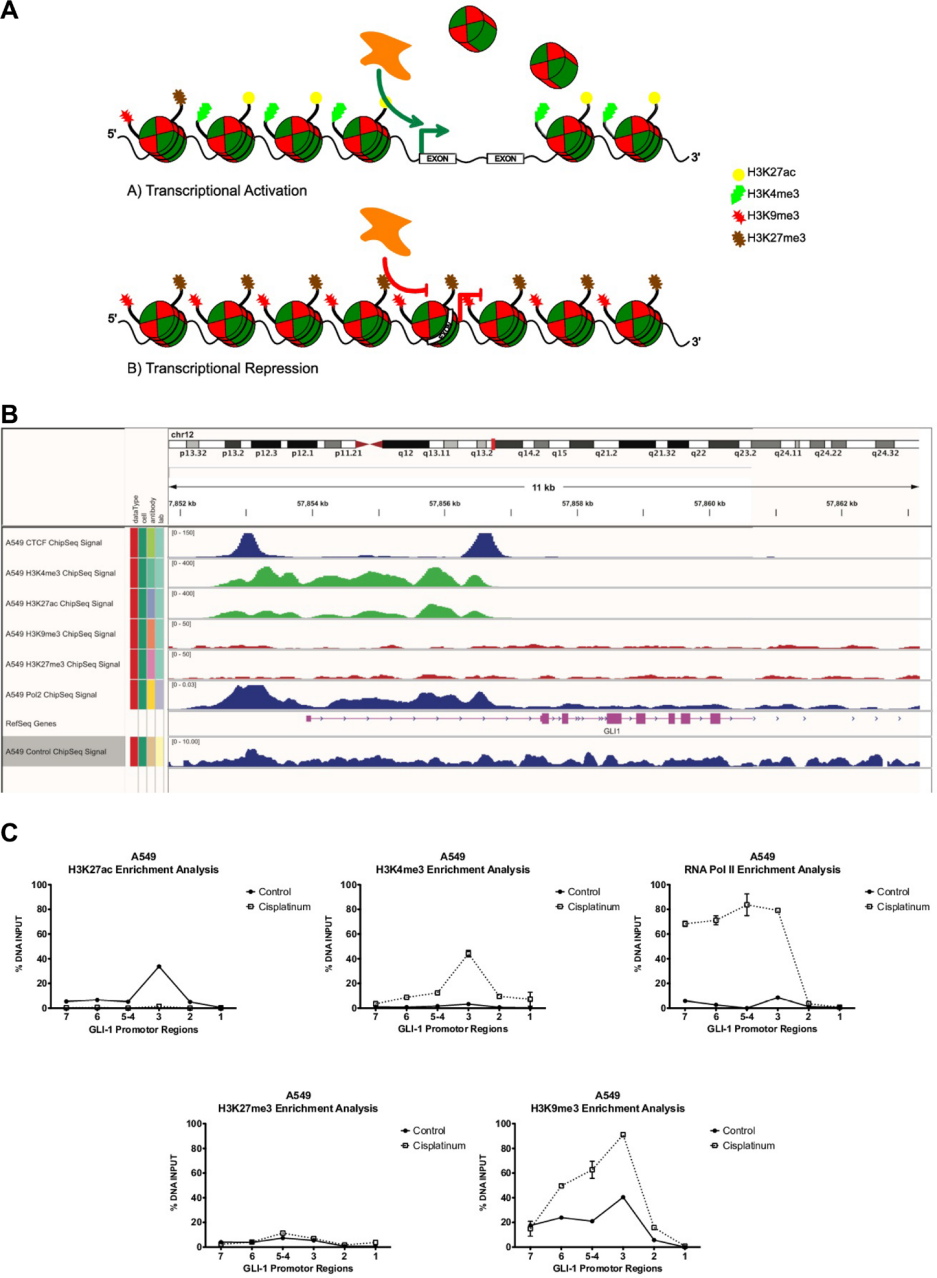
Representative model of histone code remodeling processes with or without chemotherapy schemes (**A**) Schematic representation of the genetic-epigenetic regulation of *GLI1* promoter sequences through methylation of DNA and the histone code. (**B**) Epigenome analysis based on post-translational modifications in histone lysine residues, which modulate the interaction of transcription factors upon promoter regions, in euchromatin (H3K27ac and H3K4me3) or heterochromatin state (H3K9me3 y H3K27me3) and active transcription by RNA pol II. (**C**) Lung cancer A549 cells and their histone code on the GLI1 promoter region, in presence of pharmacological challenge with cisplatin 8 μM at 48 hours, an enrichment of the activation marking H3K4me3 is seen, as well as a significant positioning of an activated RNA Pol II after the pharmacological challenge, suggesting an epigenetic reprogramming, favoring the transcriptional activity mediated by the histone code. Assays developed with the use of the real-time PCR platform Lightcycler 480 (Roche, Mannheim, Alemania), SYBR Green Master Mix (KAPA Science, Foster City, CA, U.S.A.). Antibodies used: anti-H3k27ac (No.cat.ab4729, Lot.GR71158-2), anti-H3k27me3 (No.cat.ab6002, Lot.GR77445-3), anti-H3k4me3 (No.cat.ab8580, Lot.GR68224-1), anti-H3k9me3 (No.cat.ab8898, Lot.GR47224-2), anti-RNA Pol II CTD phosphorylated (No.cat.ab5131, Lot.GR59740-1) all from ABCAM. Primer design and their genetic localization on GLI-1 promoter region: GLI1 7 region (Genome position -2192 -2009 bp) Primer sequence F:AGGCCGTGTGACATGTGATT, R:GACAGAGCGAGACTCCGTCT. GLI1 6 region (Genome position -1830 -1673 bp) Primer sequence F: TCGGACTCCTGACTTGAGGT, R: GACAGAGCGAGACTCCGTCT. GLI1 5-4 region (Genome position -1541 -1375 bp) Primer sequence F: CCAGCCTGGGCAAATAGTGA, R: TCAGAGACCCAGCTCAGTCA. GLI1 3 region (Genome position -822 -665 pb) Primer sequence F: CCCTCCAGAACTTCGAGACG, R: GGCTCTGGAAGAAGGTGAGG. GLI1 2 region (Genome position -612 -457 bp) Primer sequence F: TTCCATCCAAAGGGTGAGGC, R: CCCCGACAACCAGATTGAGG. GLI1 1 region (genome position -301 -109 bp) Primer sequence F: AAAAAATTTAGTCGTTTCGTTTGA, R: TTATTAAAACGCTACCTCCGAA.

Genomic DNA methylation analysis is based on the detection and quantification of the covalent addition of methyl groups to the fifth position of a cytosine base (5-methyl-cytosine “5mC”) or of demethylation in the context of CpG islands. This phenomenon depends on the orchestrated action of specific proteins with methylated-DNA binding sites (MBD) as well as the enzymatic action of DNA methyltransferases on promoter sequences or control regions, which permit activation *vs* repression of gene expression. The repercussions of post-translational modifications on the histone code have also been studied, which include effects on the condensation level of chromatin as well as the nucleosome structure. Consequentially, the modifications affect the mechanisms of genetic replication [102] and the transcriptional rate of gene expression (Figure 4A, 4B), generating wide effects on complex biological systems, superior organisms, and human oncologic diseases [103–105].

Experimental evidence has been scarce regarding members of the Hh pathway; nonetheless, hypermethylation patterns have been observed on promoter DNA sequences of PTCH1 in astrocytoma and medulloblastoma cell lines, which correlate with an excessive activation of the Hh pathway, favoring the maintenance and progression of the neoplasm [106, 107]. In a study of 112 BCC, it was also proven that PTCH1 and APC promoter sequences show hypermethylation when compared with 124 non-neoplastic skin tissues [107].

Additionally, it has been demonstrated that activation of the Hh pathway modifies the genetic expression of DNA methyltransferases DNMT1 and DNMT3a in pancreatic cancer, showing that after inhibition with cyclopamine and interference RNA against GLI-1 transcripts, mRNA and protein levels of DNMTs decrease. In contrast, when GLI-1 is overexpressed, mRNA and protein levels increase, promoting epigenetic changes through DNA methylation of CpG islands [108].

GLI-1 has been shown to be able to bind to promoter sequences of the DNMT1 gene (an enzyme in charge of maintaining DNA methylation patterns), in contrast to DNMT3a and DNMT3b (which are involved in *de novo* methylation). This shows how GLI-1 overexpression promotes an increase in methylation levels of genes such as APC in pancreatic cancer [108, 109]. Evidence from primary cultures of medulloblastoma, as well as *in vivo* models, has also shown that sustained activation of the Hh pathway in turn increases expression levels of HDACs, affecting the remodeling of activation states of chromatin. In this case, the increases in mRNA and protein levels of members of the HDAC family suggest, and at the same time confirm, that these are required as molecular beacons of survival and/or progression in tumor cells [110]; nonetheless, post-translational regulation mechanisms are also recognized.

### Post-translational regulation of the Hh pathway

Scarce reports regarding the post-translational and/or epigenetic regulation mechanisms involved in the Hh pathway have been published which are based on the activity of non-coding RNAs. In this regard, inflammatory processes in murine models with overexpression of the inducible nitric oxide synthase (iNOS) have been shown to promote the overexpression of miR-146a, negatively regulating NUMB, which negatively controls GLI-1 nuclear translocation [111].

Meanwhile, in non-solid neoplasms such as chronic myeloid leukemia (CML), the overexpression of SMO has been associated with a loss of expression of miR-136 in marrow-derived CD34+ cells, while overexpression of miR-326 decreases the expression of SMO, diminishing cell proliferation and increasing the apoptotic activity of CD34+ CML cells. In this case, restoring SMO activity would be able to reverse the effect of miR-326, which has been considered as a therapeutic target in non-solid neoplasms [112].

Lastly, recent evidence points to the participation of lncRNAs during neural development as well as in cell destiny and differentiation. In this regard, during early stages, lncRNA AK053922, which is located in the genetic locus of the GLI-3 gene, promotes specialized neural cell differentiation capabilities. This lncRNA exerts a bifunctional role through the inhibition or activation of Hh signaling, helping designate different types of neurons [113, 114]. The presence of lncRNAs in cancer has currently gained relevance because of their ability to modulate not only gene expression but also the activity of protein markers. One such case is pancreatic cancer, where it has been demonstrated that the expression levels of the lncRNA GLI1-AS, located with negative polarity to the GLI-1 promoter, negatively correlate with the expression levels of GLI-1, showing that silencing of GLI1-AS promotes the overexpression of GLI-1. The biological effect of this correlation is an increase in the cell proliferation index and *in vivo* tumor size. In contrast, the overexpression of GLI1-AS decreases the expression levels of GLI-1 as well as its target genes PTCH1 and PTCH2 and thus decreases cell proliferation. Additionally, it was demonstrated that silencing the GLI-1 protein decreases the expression of GLI1-AS, while overexpression of GLI-1 increases GLI1-AS, supporting the hypothesis of a negative/positive biofeedback loop where GLI1-AS is the genetic target of the transcriptional factor GLI-1 at the protein level; this epigenetic-transcriptional relationship contributes to the biomedical and clinical impact of the Hh pathway in the progression of human carcinomas [115].

### Transcriptional regulation of GLI-1

Previously, GLI-1 was proposed to be a transcriptional regulator of several gene targets that are involved in diverse tasks such as maintaining stem cells, cell proliferation mechanisms, and apoptosis specific to epithelial tissues. Among these, we can cite PTCH1, HNF3-beta (embryogenesis), FOXM1 (cell proliferation and differentiation), Cyclin D and Cyclin E (phase G1 of the cell cycle), and SNAIL (MET and metastases) as well as diverse oncogenes, including c-MYC [116–121]. Additionally, the binding of GLI-1 to the promoter region of SOX2 has recently been demonstrated, positively promoting its expression and in turn potentiating the self-renewal of SOX2 dependent-stem cells [122].

Epigenetic mechanisms have also been shown to participate in mechanisms of oncology therapy resistance, highlighting the hypomethylation of genomic DNA. Such case has been detected regarding the promoter sequences of the ABCG2 membrane receptor, a member of the superfamily of ABC cassette type receptors, which function as an ATP-dependent pump. These receptors regulate drug and xenobiotic transport to the cell exterior and multidrug resistance to several oncology therapeutic agents in neoplastic cells [123]. In this sense, it is evident that the Hh pathway promotes the expression of ABCG2 as GLI-1 is capable of binding to the promoter region of ABCG2, thus promoting resistance to therapy in diffuse large B-cell lymphoma [124]. It has also been demonstrated that epigenetic-transcriptional reprogramming events posterior to a pharmacological challenge (with platinum derivatives) contribute to the preservation of conditions of molecular identity in epithelial tumors that are dependent on the Hh pathway. This has been observed in the histone code enrichment of H3K4me3 and RNA Pol II activation despite the bivalent increase of the H3K9me3 mark, favoring the overexpression of GLI-1. This occurrence promotes the functional dependence of the Hh pathway in epithelial lung cancer cells (Figure 4C). Nonetheless, a complete description of the epigenetic-transcriptional mechanisms that underlie the Hh pathway, as well as their impacts in oncology therapy, is still lacking as is their relationship to oncology therapy resistance genes, which has been previously demonstrated in CNS tumors, such as medulloblastoma [125].

### Hh pathway in lung cancer

New evidence has arisen regarding the participation of the Hh pathway in cancer and the maintenance of cancer stem cells (CSCs), representing a therapeutic target for new treatment schemes based on the inhibition, at different levels, of the Hh pathway [126]. It is therefore important to consider the thorough study of the initiation, progression, invasion and metastatic processes that comprise the hallmarks of cancer, along with their genetic-epigenetic functional modulation processes. In addition to the study of excess Hh activation, which promotes the tumor microenvironment through pro-inflammatory mechanisms, angiogenesis, genome instability, mutation, resistance to cell death, energy imbalance, etc., are involved in invasion and metastasis [127]. In particular, GLI-1 and GLI-2 have both recently been described to have roles as crucial molecular indicators in the maintenance of cell proliferation and evasion of apoptosis in lung squamous cell carcinoma (LSCC). In this case, concomitant use of the SMO inhibitor GDC-0449 and the GLI-1 antagonist GANT61 is able to block DNA-protein binding and jointly decrease the expression of transcription factors GLI-1 and GLI-2, reducing the cell proliferation rate and increasing apoptosis *in vivo*, therefore proving the important role of GLI-2 in LSCC tumors. Meanwhile, the inhibition of GLI-1 begins to emerge as a new strategy for patients with this particular malignancy [128]. It is important to remark, however, that there is extensive evidence that points to a high rate of multiple resistances, and therefore the combined use of EGFR Tyrosine Kinase Inhibitors (TKIs) along with GLI-1 inhibitors is being recommended for NSCLC patients who may have an epithelial-mesenchymal transition (EMT) as a result of therapy resistance. This recommendation is of high relevance as the combined use of TKIs with SMO-GLI-1 inhibitors is currently being projected to be a high efficiency scheme that could reduce EMT tumor mechanisms in high-grade malignancies with poor prognosis, suggesting new therapeutic strategies for treating progressive lung cancer [129].

### Perspectives: The Hh pathway, EGFR-TKIs and lncRNAs in lung cancer

In current times, the comprehensive study of genetic, transcriptional and epigenetic regulation is of the utmost importance because they are fundamental to a full understanding not just cellular physiology, as well as of embryonic development processes until to the mechanisms that underlie the transformation and progression of complex diseases such as epithelial malignant neoplasms called “carcinomas”. In the last few years, the participation of the Hh pathway members in the development of diverse epithelial tumors, such as BCC, gastrointestinal, prostate, breast and, recently, lung carcinomas, has been described [127]. In addition, the use of SMO and GLI inhibitors, which negatively affect the Hh signaling pathway, has been shown to be of benefit to patients with carcinomas; nonetheless, little is known regarding the mechanisms that cause carcinomas to have a continuous dependency on the Hh cellular pathway, nor of its impact on tumor progression and therapy response to treatment schemes in patients with lung cancer [84, 91].

Currently, different research groups are working to better understand the different genetic, transcriptional and epigenetic modifications involved in the control of the Hh signaling pathway, which allow its continuous activation during the transformation, progression, and maintenance of malignant epithelial neoplasms, such as lung carcinomas. Such biomedicine work will consolidate its position when considering strategies for the early molecular diagnosis and/or better oncologic treatment response of patients with lung cancer.

The possible mechanisms involved in resistance to the first-line agents including platinum-derivatives based pharmacological compounds, as well as EGFR-TKIs treatment resistance explained in part by EGFR mutations including T790M resistance mutation, have currently necessitated the use of combined therapies based on platinum-derivatives, paclitaxel, and several TKI´s in others as erlotinib or WZ4002. All of these have proven to have synergic effects regarding toxicity and response, increasing apoptosis in patients who develop TKIs resistance [130]. In addition, cellular stress in pulmonary cancer models has been described as result of increase in the activity of Hh cell signaling pathway, in turn an increasing of cell survival, cell growth and invasion have been described through cell signaling by HGF and MET proteins, as well as through the Hedgehog-Interacting Protein (HHIP), whose membrane surface protein acts as a negative regulator of the Hh signaling pathway. Based on the overexpression of HHIP, as well as, use of inhibitors of the MET signaling pathway, sensitize lung cancer cells undergoing TKI-Gefitinib-based treatment [131]. In NSCLC, blocking the Hh signaling pathway using the SMO antagonist (SANT-1) restores the expression of E-cadherin; meanwhile, it decreases the expression of Snail and ABCG2 in EGFR-TKIs resistant NSCLC cells. As such, the combined use of SANT-1 and Gefitinib reduce tumorigenesis and cell proliferation in EGFR-TKI resistant cells, thus confirming that blocking the Hh signaling pathway constitute a synergistic mechanism for the sensitization in front to EGFR-TKIs treatments in NSCLC cells [132].

In addition, novel transcriptional and epigenetic control mechanisms for the Hh cell signaling pathway has also recently been described. About that, mechanisms relies on Homeobox-type transcription factors overexpressed during the embryonic segmentation and differentiation of mesenchymal layers, such as Mesenchyme Homeobox-2 factor, have been detected controlling to the GLI-1 gene expression at gene promoter level, promoting lung oncology therapy resistance capacity, and also been associated with progression, global survival and response capacity to therapy in lung cancer patients [133]. These findings have represented an important contribution to the molecular epigenetic-transcriptional understanding of the Hh pathway regulation for the clinical application in patients with lung cancer.

Based above mentioned, and as probable epigenetic mechanisms involved, it is important to discuss the overexpression and function of lncRNAs, such as UCA1 that are involved in the resistance of lung cancer cells to Gefitinib (PC9/R and H1975), as well as, in lung cancer patients with acquired resistance to EGFR-TKI therapy with deletion of exon19 and/or genetic mutation of exon21 (L858R), where UCA1 has been associated with poor progression free survival in patients free from T790M mutation status [134].

**Figure 5:**
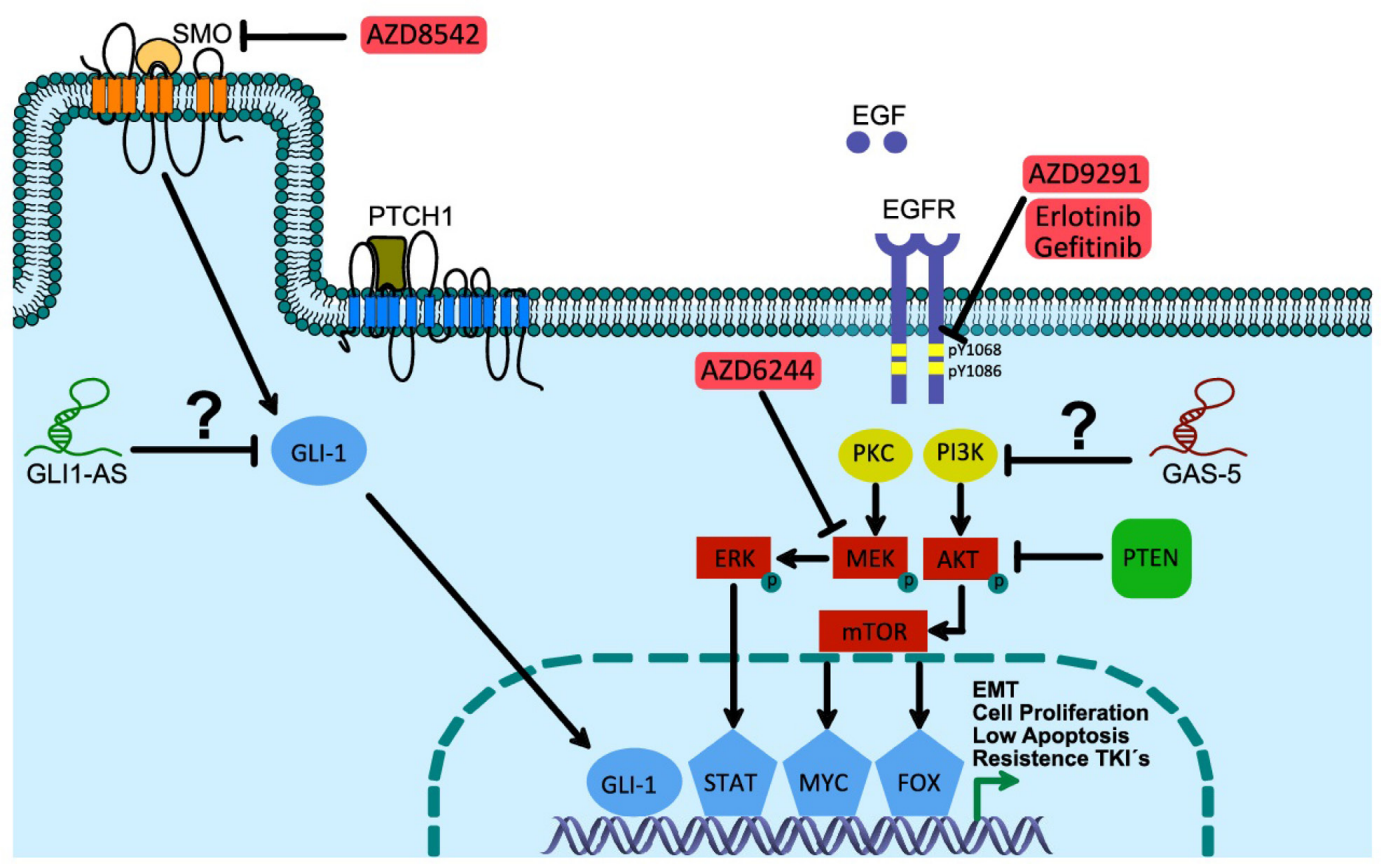
Hedgehog and EGFR cell signaling pathways: under lncRNAs control in lung cancer cells? SMO-antagonist compound AZD8542 negatively controls Hh pathway activation, promoting the genetic expression and/or activation of GLI-1. Additionally, it has been recently observed that an association exists between lncRNA GLI1-AS and GLI-1 mRNA expression levels, and afterwards promoting GLI-1 displacement to the cell membrane for a posterior interaction with an endogenous ligand (a GTPase type protein has been proposed). This promotes the nuclear translocation and transcriptional activity of GLI1^A^. The absence of SHH maintains the interaction of PTCH1 with SMO, contributing to the inhibition by blocking its displacement to the cell membrane, and promoting GLI proteolysis, which translocates as GLIR, and modulates the transcriptional repression of, among others, PTCH1, Cyclin D and E, FOXM1, ABCG2, and SOX2 genes.

On that a probable connection between the efficacy of the EGFR-TKI-related therapy and overexpression of the lncRNA SOX2-OT and SOX2 protein has been associated with lung cancer cell proliferation and poor survival in a cohort that included 83 lung cancer patients [135]. In addition to a probable transcriptional biofeedback of the GLI-1 protein, in solid epithelial tumors, overexpression of the lncRNA GLI1-AS and loss of expression of the lncRNA GAS5 are both probably involved in resistance to oncology therapy in lung cancer (Figure 5) [115].

In conclusion, based on all above described information, it is necessary to contemplate the combined use of drugs in epigenetic and posttranscriptional reprogramming strategies, such as lncRNAs combined with specific inhibitors of the Hh pathway. Additionally, elevating therapeutic efficiency by using drugs directed against genes that code for chromatin remodeling and/or methylation in promoter regions and decrease the activity of chemoresistance will offer lung cancer patients a better quality of life.
